# Physical Activity Behavior of Patients at a Skilled Nursing Facility: Longitudinal Cohort Study

**DOI:** 10.2196/23887

**Published:** 2022-05-23

**Authors:** Ramin Ramezani, Wenhao Zhang, Pamela Roberts, John Shen, David Elashoff, Zhuoer Xie, Annette Stanton, Michelle Eslami, Neil S Wenger, Jacqueline Trent, Antonia Petruse, Amelia Weldon, Andy Ascencio, Majid Sarrafzadeh, Arash Naeim

**Affiliations:** 1 Center for Smart Health University of California, Los Angeles Los Angeles, CA United States; 2 Department of Computer Science University of California, Los Angeles Los Angeles, CA United States; 3 Department of Physical Medicine and Rehabilitation Cedars-Sinai Medical Center Los Angeles, CA United States; 4 Department of Biomedical Sciences Cedars-Sinai Medical Center Los Angeles, CA United States; 5 Department of Hematology and Oncology University of California, Los Angeles Los Angeles, CA United States; 6 Department of Medicine Statistics Core, Biostatistics and Computational Biology University of California, Los Angeles Los Angeles, CA United States; 7 Department of Psychology University of California, Los Angeles Los Angeles, CA United States; 8 Rockport Healthcare Services Los Angeles, CA United States; 9 Division of General Internal Medicine University of California, Los Angeles Los Angeles, CA United States

**Keywords:** physical medicine and rehabilitation, geriatrics, remote sensing technology, physical activity, frailty, health care delivery models, wearable sensors, indoor localization, Bluetooth low energy beacons, smartwatches

## Abstract

**Background:**

On-body wearable sensors have been used to predict adverse outcomes such as hospitalizations or fall, thereby enabling clinicians to develop better intervention guidelines and personalized models of care to prevent harmful outcomes. In our previous work, we introduced a generic remote patient monitoring framework (Sensing At-Risk Population) that draws on the classification of human movements using a 3-axial accelerometer and the extraction of indoor localization using Bluetooth low energy beacons, in concert. Using the same framework, this paper addresses the longitudinal analyses of a group of patients in a skilled nursing facility. We try to investigate if the metrics derived from a remote patient monitoring system comprised of physical activity and indoor localization sensors, as well as their association with therapist assessments, provide additional insight into the recovery process of patients receiving rehabilitation.

**Objective:**

The aim of this paper is twofold: (1) to observe longitudinal changes of sensor-based physical activity and indoor localization features of patients receiving rehabilitation at a skilled nursing facility and (2) to investigate if the sensor-based longitudinal changes can complement patients’ changes captured by therapist assessments over the course of rehabilitation in the skilled nursing facility.

**Methods:**

From June 2016 to November 2017, patients were recruited after admission to a subacute rehabilitation center in Los Angeles, CA. Longitudinal cohort study of patients at a skilled nursing facility was followed over the course of 21 days. At the time of discharge from the skilled nursing facility, the patients were either readmitted to the hospital for continued care or discharged to a community setting. A longitudinal study of the physical therapy, occupational therapy, and sensor-based data assessments was performed. A generalized linear mixed model was used to find associations between functional measures with sensor-based features. Occupational therapy and physical therapy assessments were performed at the time of admission and once a week during the skilled nursing facility admission.

**Results:**

Of the 110 individuals in the analytic sample with mean age of 79.4 (SD 5.9) years, 79 (72%) were female and 31 (28%) were male participants. The energy intensity of an individual while in the therapy area was positively associated with transfer activities (β=.22; SE 0.08; *P*=.02). Sitting energy intensity showed positive association with transfer activities (β=.16; SE 0.07; *P*=.02). Lying down energy intensity was negatively associated with hygiene activities (β=–.27; SE 0.14; *P*=.04). The interaction of sitting energy intensity with time (β=–.13; SE 0.06; *P*=.04) was associated with toileting activities.

**Conclusions:**

This study demonstrates that a combination of indoor localization and physical activity tracking produces a series of features, a subset of which can provide crucial information to the story line of daily and longitudinal activity patterns of patients receiving rehabilitation at a skilled nursing facility. The findings suggest that detecting physical activity changes within locations may offer some insight into better characterizing patients’ progress or decline.

## Introduction

The population aged 65 years and older is projected to double in size to 83.7 million by 2050 only in the United States [1]. With the increase in the geriatric population, health care use is expected to increase drastically with the concomitant demand for rehabilitation and in-home care after hospitalization. Many hospitalized older adults are discharged with new or worse participation in activities of daily living (ADL). Identification of patients’ unmet ADL needs in terms of functional status at the time of discharge and after they return home could help address vulnerabilities prior to hospital discharge. Functional disability, prevalent among geriatrics, is a multidimensional concept that involves factors reflected in a person’s basic actions including mobility, ADL, cognition, and vision. Whether a patient has sufficient ability to perform their ADL and mobility can be a predictor of whether they are able to remain in the community. Functional status is an important predictor of health outcomes, and emphasis on better quantifying it and understanding its limitations over longer periods of time is warranted [2-5].

In rehabilitation settings, patients work with physical and occupational therapists depending on their disability. Their functional status is measured by standardized scales to evaluate impaired motor functions, limitations in performing daily activities, reaching, grasping capabilities, and so on. While such scales may not always fully capture the motor functions, completion of a task by patients may also not always reflect improvement in motor functions in that patients learn to adopt different “synergistic patterns to compensate for lost functions” [2]. In such scenarios, physical activity wearable sensors can provide quantifiable and accurate measures of human body movements through which the effect of an injury or a disease on the movement system can be investigated. However, despite the widespread use of such technologies, their clinical use has yet to translate from “bench to bedside” [2-16].

With the advent of commercially available low-cost and lightweight sensors over the past decade, the development of remote health monitoring systems has been extensively fostered and largely investigated as a tool to provide constant vigilance to patients. Their portability and ease of use make them widely practical and applicable in a variety of living settings, providing a comprehensive illustration of activities of daily living for patients living with mobility deficits as well as healthy individuals.

In a previous study [16] we reported on the performance of our developed remote monitoring system, Sensing At-Risk Population (SARP), which is comprised of activity tracking wearable sensors and indoor localization sensors. We monitored the first 3 days of patients in subacute rehabilitation environment (baseline) using SARP. This paper extends that analysis by looking at the longitudinal data captured by SARP system in a skilled nursing facility. The goal of our analysis was to determine if longitudinal changes of sensor-based physical activity and indoor localization features of patients receiving rehabilitation can complement changes captured by therapist assessments over the course of rehabilitation in the skilled nursing facility.

## Methods

### Participants

From June 2016 to November 2017, patients were recruited after admission to a subacute rehabilitation center in Los Angeles. A longitudinal study of the physical therapy, occupational therapy, and sensor-based data assessments was performed. The study cohort contains patients admitted to a skilled nursing facility for an intended rehabilitation course of no more than 21 days. After this period, patients were either re-admitted to hospital or stayed in the community or in their residence in long-term care.

Participants were eligible if older than 60 years of age, English speaking, and able to sign a consent form approved by University of California, Los Angeles, Institutional Review Board (IRB# 16-000166 entitled Sensing in At-Risk Populations). Exclusion criteria were movement disorders or complete paralysis of the upper or lower extremities. The diversity of cohort comprised patients who were postsurgical and poststroke and had functional limitations because of medical illnesses.

### Study Design

Patients were given a smartwatch every morning at 9 am, and the watches were collected from them at around 6 PM daily. Sensors placed throughout the facility collected data passively without any interaction required from patients. Patients normally stayed in the *resident room* (bedroom) and were scheduled for an hour of daily exercise and activity in the *therapy area of the nursing home*.

### SARP System Overview

**Figure 1:**
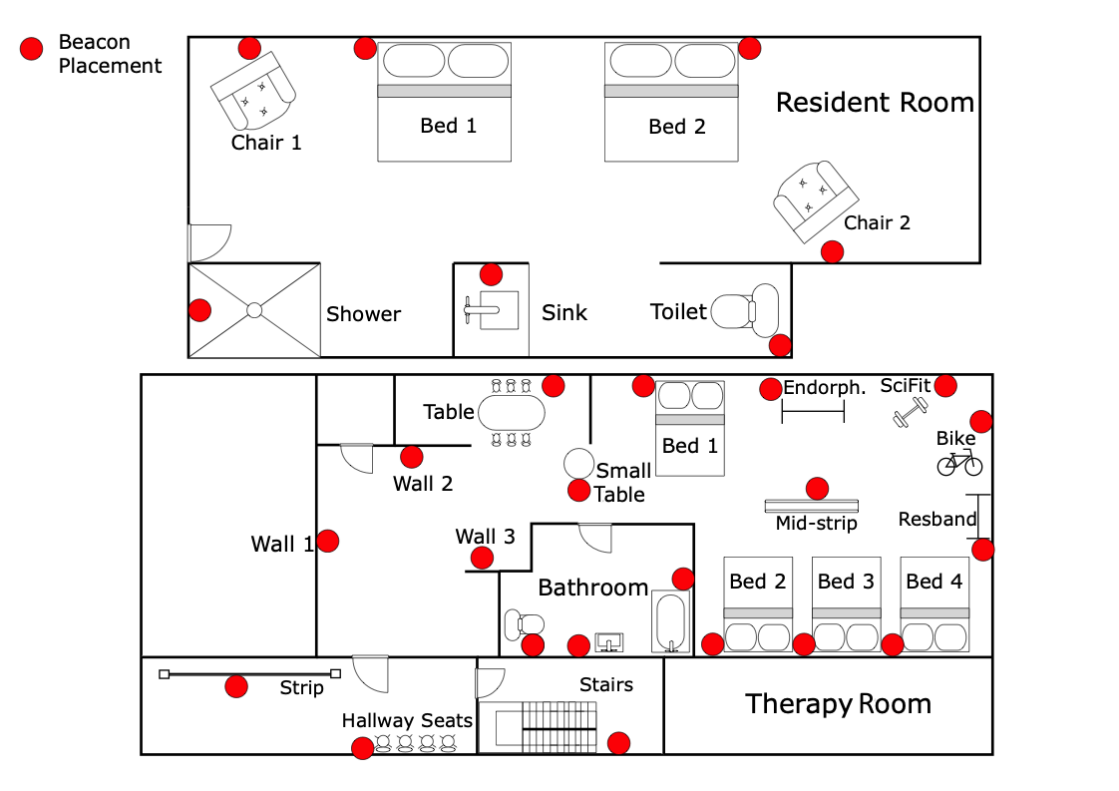
Skilled nursing facility map with beacon placements shown with red dots [16].

The core of SARP is comprised of the following: hardware—(1) commercially available Sony SmartWatch 3 with built-in EM7180 ± 2 g triaxial accelerometer, 420mA battery, and BCM43340 Bluetooth module; (2) proximity beacons (MCU ARM Cortex-M4 32-bit processor) mounted at *locations of interest* within resident rooms (bedrooms) and therapy area, shown with red color dots in Figure 1; clinically validated software—activity recognition, indoor localization, and data visualization algorithms, all encompassed within a Health Insurance Portability and Accountability Act–compliant infrastructure.

Details of the system architecture can be found in [16-20], and the patent is described in [21]. Activity tracking and indoor localization models were built, validated, and refined prior to this study on a separate cohort of patients [17].

### Measures

#### Clinical Features

Clinical assessments in this study are 2-fold: physical therapy (PT) and occupational therapy (OT). PT and OT metrics included functional activities such as bed mobility (includes rolling, moving between supine and sitting, scooting in supine, scooting on the edge of the bed), gait (movement patterns that make up walking and associated interpretations), transfers (moving body from one surface to another without walking), hygiene, toileting, and lower body dressing. Those activities were scored based on the functional levels (1 to 6), from independent to completely dependent [22]. A comprehensive collection of PT and OT key metrics were performed every week; hence, patients were expected to have ≥3 PT or OT assessments within 21 days. In this study, a subset of clinical features was chosen; these features were common in more than 65% (n=72) of patients’ PT and OT visits. The most common PT functional activities, performed by more than 65% of the cohort, are as follows: gait distance (in feet), transfer activity, and bed mobility, including movement from supine to sit. Common OT functional activities are comprised of lower body dressing, toileting activity, hygiene, and overall ability to tolerate daily activities (activity tolerance).

#### Sensor-Based Features

Time and frequency domain characteristics of the accelerometer signal (main, median, variance, skewness, kurtosis, peak frequency, and peak power) were used to determine physical activities. Indoor localization was achieved by using beacons mounted on locations of interest.

The metrics captured from smartwatches and beacons were used to infer the following features: (1) activity recognition measures such as sitting time and standing time; (2) indoor localizations, such as time in bed, time in the bathroom, or therapy area; and (3) raw acceleration quantification (ie, mean absolute deviation, which is approximately equal to energy spent). By combining these attributes, we achieved features such as sitting time in bed, energy spent while walking, lying down time in bed, and so on. Equations resulted in sensor-based feature quantifications can be found in Table 1.

To simplify the result and avoid unnecessary complexity, we focused on the most comprehensive and significant sensor-based feature (ie, energy intensity trends), consistent with analysis shown in [16].

**Table 1:** Sensor-based features.

Number	Equation	Summary
(1)	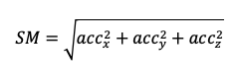	Signal magnitude
(2)	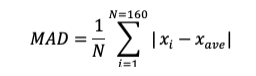	MAD^a^ of accelerometer magnitude signal≈energy spent
(3)	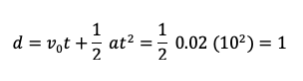	Hand displacement in 10 s when threshold on MAD=0.02 m/s^2^
(4)	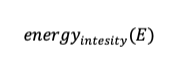	Energy spent in *walking, sitting, standing, laying,* or in *locations of interest* divided by their corresponding *time spent*. In addition to energy intensity spent at each location, we calculated the total energy intensity in *resident* room and *therapy* room. 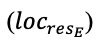 is 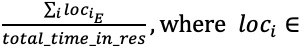 resident room. Energy intensity for therapy room was similarly calculated.

^a^MAD: mean absolute deviation.

### Analysis Inclusion Criteria

Analysis inclusion criteria were defined to ensure all patients satisfy a minimum amount of daily sensor data and collected PT and OT assessments. Analysis criteria include patients with the following data: (1) ≥3 days of watch data; (2) each day ≥4 hours of watch wear time; and (3) ≥3 sessions of PT or OT or a combination of both PT and OT.

**Figure 2:**
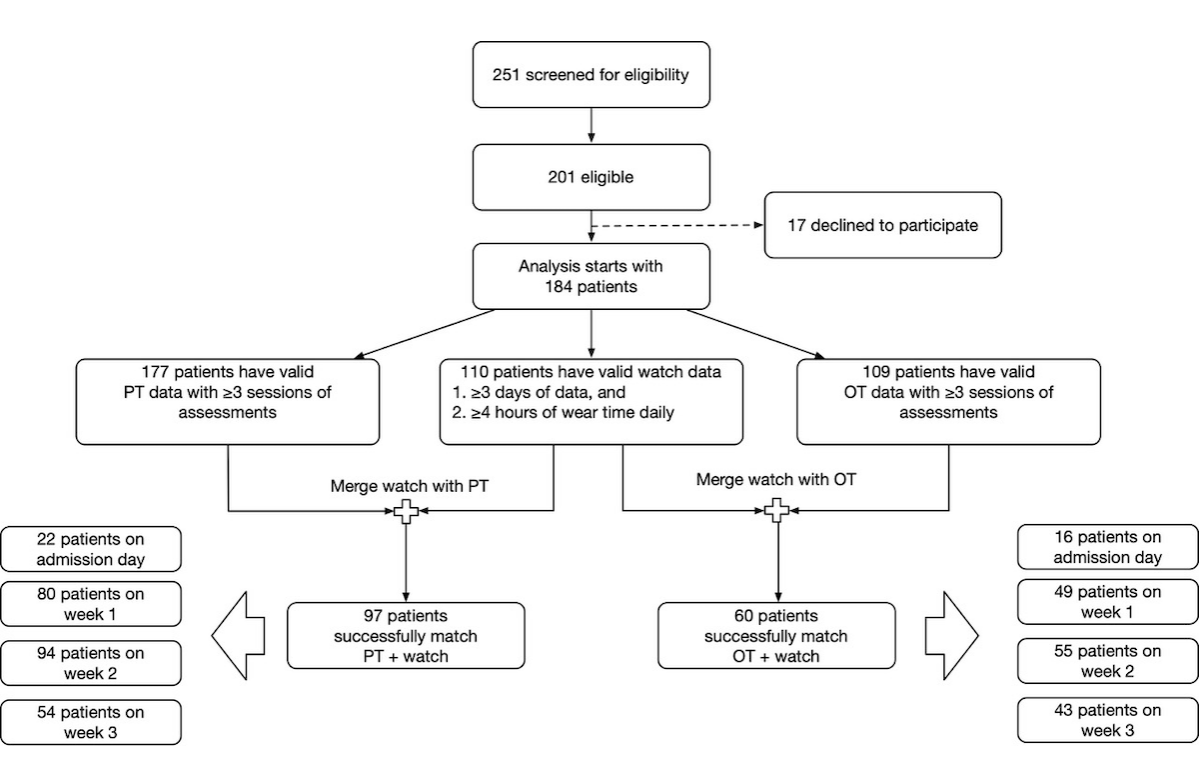
Diagram describing the analysis cohort. OT: occupational therapy; PT: physical therapy.

Cohort data were agglomerated for analyses according to the consort diagram shown in Figure 2.

### Statistical Analyses

Visualization of prior analysis was generated to unveil any longitudinal patterns. The time trends of sensor-based features appeared to be approximately linear; hence, we decided to use linear models for longitudinal analysis.

Descriptive statistics (medians and IQR) were computed for clinical assessments (ie, PT and OT) at each session. Generalized linear mixed effect model was used to understand the longitudinal relationships between the clinical measures and the sensor-based features [23-26]. Due to the frequency difference in which sensor and clinical assessments were collected, we merged a day of clinical assessment data with its corresponding day or closest day containing the sensor data (SD 3 days). Note that a valid day of sensor data should satisfy the analysis inclusion criteria 1 and 2.

Three models, each with different sets of sensor-based features, were constructed for each clinical outcome. Model 1 included overall energy intensity as covariate. Model 2 considered energy intensity at resident room and energy intensity at therapy area as covariates. Additionally, sensor-based activity parameters (eg, energy intensity of sitting) were used in model 3. Linear time indicates the number of weeks since the enrollment day. Interaction effects of sensor features with time were also included.

### Ethics Approval

The Ethics Board reviewed this study. The following was their determination: “The UCLA Institutional Review Board (UCLA IRB) has approved IRB#16-000166 entitled ‘Sensing At Risk Populations (SARP).’ UCLA's Federal wide Assurance (FWA) with Department of Health and Human Services is FWA00004642. The UCLA IRB waived the requirement for HIPAA Research Authorization to identify potential research participants. The UCLA IRB waived the requirement for informed consent for the review of medical records to identify potential research participants under 45 CFR 46.116(d). The UCLA IRB waived the requirement for signed informed consent for participants admitted to the BECH for acute care under 45 CFR 46.117(2).”

## Results

### Demographic Analysis

From 184 consented patients, 110 (60%) met the watch wearing time protocol with mean age of 79.4 (SD 5.9) years. Moreover, 97 (88%) patients were included in PT-watch paired analysis and 60 (54%) in OT with watch analytics. Most participants were female (n=79, 72%) and of White race or ethnicity (n=84, 76%). Additionally, 62% (n=69) of the patients had pain, 99% (n=109) of them needed some level of assistance with functional mobility activities (transfer activity), and 75% (n=83) needed assistive devices for walking. Table 2 presents detailed sociodemographic and clinical characteristics of the 110 patients. ADL parameters and their significance in determining the outcome are presented based on initial assessments, at the time of admission, or within one day.

**Table 2:** Sociodemographic and clinical characteristics (initial assessment) of the cohort of 110 patients.

Parameters		Community	Hospital	Parameter discriminative power (*P* value)
Subject, n (%)		105 (95.5)	5 (4.5)	N/A^a^
Age (years), mean (SD)		78.0 (5.7)	84.1 (6.8)	.03
**Gender, n (%)**				>.99
	Female	76 (72.4)	3 (60)	
	Male	29 (27.6)	2 (40)	
**Race or ethnicity, n (%)**				>.99
	Asian	5 (4.8)	0 (0)	
	Black or African American	12 (11.4)	1 (20)	
	Hispanic or Latino	2 (1.9)	0 (0)	
	Native or Hawaiian Pacific Islander	2 (1.9)	0 (0)	
	White	84 (80)	4 (80)	
**Pain present, n (%)**				.95
	No	29 (30)	2 (50)	
	Yes	67 (70)	2 (50)	
**Active diagnoses, n (%)**				.86
	<10	22 (21)	0 (0)	
	≥10	83 (79)	5 (100)	
**Transfers, n (%)^b^**				.87
	Supervision	1 (1)	0 (0)	
	Limited assistance	57 (55)	1 (20)	
	Extensive assistance	46 (44)	4 (80)	
**Dressing, lower body, n (%)**				.93
	Independent	1 (1)	0 (0)	
	Limited assistance	28 (27)	0 (0)	
	Extensive assistance	75 (72)	5 (100)	
**Eating, n (%)**				.93
	Independent	94 (90)	4 (80)	
	Supervision	4 (4)	1 (20)	
	Limited assistance	4 (4)	0 (0)	
	Extensive assistance	2 (2)	0 (0)	
**Toileting, n (%)**				.70
	Independent	1 (1)	0 (0)	
	Limited assistance	45 (43)	0 (0)	
	Extensive assistance	58 (56)	5 (100)	
**Walk room, n (%)**				.91
	Supervision	1 (1)	0 (0)	
	Limited assistance	61 (59)	1 (20)	
	Extensive assistance	34 (32)	3 (60)	
	Activity did not occur	8 (8)	1 (20)	
**Walk hall, n (%)**				.92
	Supervision	1 (1)	0 (0)	
	Limited assistance	62 (60)	1 (20)	
	Extensive assistance	35 (33)	4 (80)	
	Activity occurred only once or twice	1 (1)	0 (0)	
	Activity did not occur	5 (5)	0 (0)	
**Walk on unit, n (%)**				.78
	Supervision	1 (1)	0 (0)	
	Limited assistance	62 (60)	1 (20)	
	Extensive assistance	41 (39)	4 (80)	
**Hygiene, n (%)**				.84
	Independent	1 (1)	0 (0)	
	Limited assistance	59 (57)	2 (40)	
	Extensive assistance	44 (42)	3 (60)	
**Bed mobility, n (%)**				.96
	Supervision	1 (1)	0 (0)	
	Limited assistance	68 (65)	2 (40)	
	Extensive assistance	35 (34)	3 (60)	
**Urinary continence, n (%)^b^**				.002
	Always continent	85 (82)	1 (20)	
	Occasionally incontinent	3 (3)	0 (0)	
	Frequently incontinent	7 (6)	1 (20)	
	Always incontinent	4 (4)	3 (60)	
	Not rated	5 (5)	0 (0)	
**Bowel continence, n (%)^b^**				.006
	Always continent	91 (87)	2 (40)	
	Occasionally incontinent	3 (3)	0 (0)	
	Frequently incontinent	5 (5)	0 (0)	
	Always incontinent	5 (5)	3 (60)	
**Assistive devices, n (%)**				>.99
	Wheelchair	3 (4)	0 (0)	
	Walker and wheelchair	75 (95)	4 (100)	
	Cane and wheelchair	1 (1)	0 (0)	

^a^N/A: not applicable.

^b^Parameters with *P*<.05.

### Longitudinal Analysis of All Features (Sensor and Clinical Measurements)

The community group spent higher overall energy intensity and energy intensity at the resident room compared to the hospital group, as seen in Figures S1 (a) and S1 (b) of Multimedia Appendix 1. However, energy intensity during therapy sessions tends to have similar values between two groups, especially toward the end of the rehabilitation period, as seen in Figure S1 (c) of Multimedia Appendix 1.

The descriptive statistics of clinical parameters are summarized in Table 3. It shows that “gait distance feet” increases over time (median and IQR after the first week), and “activity tolerance” increases (IQR after first week and median after second week). The table indicates no clear improvements in other clinical-based measures gauged by PT and OT functional levels within 3 weeks.

**Table 3:** Descriptive statistics of all measures.

Measures			Admission day						Week 1							Week 2							Week 3				
			N		Median		IQR		N		Median		IQR		N			Median		IQR		N			Median		IQR
**Sensor features**																											
	Overall_EI^a^	110		17.97		13.00~23.74		110		18.88		13.76~25.17		83			19.30		14.97~25.05		57			18.43		15.10~23.37	
	Resident_room_EI	110		19.41		14.90~24.74		110		19.94		15.58~25.85		83			20.65		16.12~25.66		57			19.45		15.69~24.39	
	Therapy_room_EI	110		15.09		9.02~25.36		110		15.29		9.83~25.01		83			17.19		10.30~24.34		57			14.96		11.20~20.50	
**Occupational therapy features**																											
	Dressing, lower body	16		3.00		2.75~3.00		39		4.00		3.00~4.00		40			4.00		4.00~4.00		31			4.00		4.00~4.00	
	Toileting general	16		4.00		2.75~4.00		37		4.00		3.00~4.00		40			4.00		4.00~4.00		29			4.00		4.00~4.00	
	Activity tolerance general (min)	11		8.00		5.00~9.00		34		15.00		10.00~15.00		37			15.00		15.00~20.00		29			20.00		15.00~20.00	
	Hygiene grooming	4		4		4.00~4.00		15		4.00		4.00~4.00		19			4.00		4.00~4.00		15			4.00		4.00~4.00	
**Physical therapy features**																											
	Transfer general	20		4.00		3.75~4.00		72		4.00		4.00~4.00		86			4.00		4.00~4.00		50			4.00		4.00~4.00	
	Gait distance, feet	20		40.00		18.75~50.00		70		100.00		71.25~150.00		80			150.00		100.00~200.00		44			150.00		97.50~200.00	
	Gait assistive device	21		2.00		1.00~2.00		60		2.00		2.00~2.00		69			2.00		2.00~2.00		38			2.00		2.00~2.00	
	Gait level surface	18		4.00		4.00~4.00		61		4.00		4.00~4.00		71			4.00		4.00~4.00		40			4.00		4.00~4.00	
	Bed mobility supine sit	21		4.00		3.00~4.00		72		4.00		4.00~4.00		84			4.00		4.00~4.00		49			4.00		4.00~4.00	

^a^EI: energy intensity.

### Longitudinal Association Between Clinical Measures and Sensor-Based Features

The associations of repeated PT, OT, and sensor-based measurements are modeled through three generalized linear mixed models. On PT and sensor associations, according to Table 4, the results of model 1 revealed that gait distance feet (β=.28; SE=0.06; *P*<.001), gait level surface β=.17; SE=0.04; *P*<.001, and bed mobility including supine to sit (β=.26; SE=0.05; *P*<.001) improved over time. Higher overall energy intensity indicates a higher score of transfer activity (β=.22; SE=0.08; *P*=.03).

In model 2, energy intensity at the therapy room was positively associated with transfer activity (β=.19; SE=0.08; *P*=.02). In addition, gait distance feet (β=.28; SE=0.05; *P*<.001), gait level surface (β=.17; SE=0.04; *P*<.001) and bed mobility including supine to sit (β=.26; SE=0.05; *P*<.001) improved every week.

In model 3, sitting energy intensity showed positive association with transfer activity (β=.16; SE=0.07; *P*=.02). Meanwhile, according to model 3, participants showed weekly improvements in gait distance (measured in feet; β=.27; SE=0.06; *P*<.001), gait level surface (β=.16; SE=0.05; *P*<.001), and bed mobility including supine to sit (β=.26; SE=0.05; *P*<.001).

On OT and sensor associations, Table 4 shows that lower body dressing, toileting activity, and activity tolerance in general improved every week in all three models. The higher value of overall energy intensity in model 1 implied a higher functional score of lower body dressing (β=.19; SE=0.09; *P*=.03) and toileting activity (β=.23; SE=0.09; *P*=.01).

**Table 4:** Generalized linear mixed model association between physical therapy and occupational therapy assessments with sensor-based features.

Models			Gait distance feet					Transfer general					Gait level surfaces					Bed mobility supine sit					Dressing lower body					Toileting general					Activity tolerance general		
			Estimate β		SE		Estimate β			SE		Estimate β			SE		Estimate β			SE		Estimate β			SE		Estimate β			SE		Estimate β			SE
**Model 1**																																			
	Intercept	–.01		0.09		–.01			0.09		.02			0.11		.01			0.09		<.01			0.10		.01			0.13		<.01			0.10	
	Time (weeks)	.28		0.06^a^		.08			0.05		.17			0.04^a^		.26			0.05^a^		.30			0.07^a^		.16			0.05^b^		.59			0.06^a^	
	Overall EI^c^	.14		0.08		.22			0.08^b^		.11			0.08		.18			0.08^b^		.19			0.09^b^		.23			0.09^b^		–.08			0.08	
	Time × overall EI	.01		0.06		–.05			0.05		–.07			0.05		–.09			0.05		–.09			0.07		–.04			0.06		–.01			0.07	
**Model 2**																																			
	Intercept	<–.01		0.08		–.02			0.09		.01			0.10		.01			0.09		<–.01			0.10		.01			0.13		<.01			0.10	
	Time (weeks)	.28		0.05^a^		.08			0.05		.17			0.04^a^		.26			0.05^a^		.29			0.07^a^		.15			0.05^b^		.59			0.06^a^	
	Resident room EI	.16		0.10		.06			0.09		.02			0.10		.14			0.09		.07			0.10		.14			0.10		.04			0.29	
	Therapy room EI	–.05		0.08		.19			0.08^b^		.10			0.08		.07			0.07		.16			0.10		.15			0.08		–.02			0.24	
	Resident room EI × time	.07		0.07		–.04			0.07		.01			0.06		–.08			0.06		–.07			0.09		–.06			0.07		–.02			0.12	
	Therapy room EI × time	–.08		0.07		.02			0.07		–.10			0.06		–.01			0.06		.02			0.09		.05			0.08		–.01			0.10	
**Model 3**																																			
	Intercept	–.01		0.08		–.01			0.09		.02			0.11		.01			0.09		–.01			0.11		.02			0.14		<.01			0.10	
	Time (weeks)	.27		0.06^a^		.06			0.05		.16			0.05^a^		.26			0.05^a^		.32			0.07^a^		.18			0.05^a^		.59			0.06^a^	
	Sitting EI	.03		0.07		.16			0.07^b^		.03			0.06		<.01			0.06		.13			0.09		.09			0.07		.10			0.07	
	Standing EI	–.01		0.09		.06			0.08		.07			0.07		–.03			0.08		.07			0.11		.03			0.08		–.03			0.09	
	Laying down EI	.13		0.09		.06			0.09		.06			0.08		.14			0.08		.03			0.11		.10			0.11		–.14			0.09	
	Sitting EI × time	.03		0.06		–.04			0.05		–.01			0.05		–.02			0.05		–.15			0.08		–.13			0.06^b^		–.13			0.07	
	Standing EI × time	.08		0.07		.11			0.07		.02			0.06		.04			0.06		–.05			0.10		–.07			0.07		.04			0.09	
	Laying down EI × time	–.01		0.08		–.13			0.07		–.09			0.06		–.09			0.07		.11			0.11		.15			0.09		–.10			0.08	

^a^*P*<.001.

^b^*P*<.05.

^c^EI: energy intensity.

### Longitudinal Analyses of Location Occurrences Between 2 Outcome Categories of Patients

**Figure 3:**
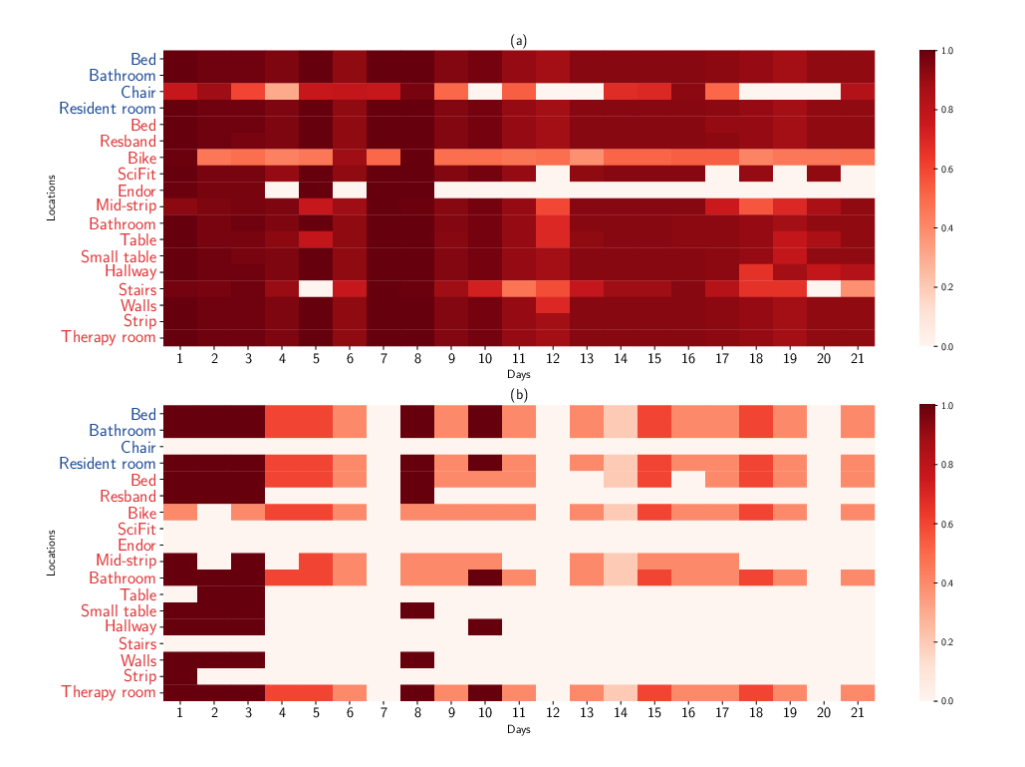
Normalized observation counts per patient by location within 21 days; (a): 105 patients in the "community" group; (b): 5 patients in the "hospital" group.

The occurrence of a location is equal to the number of times a patient spends more than 40 continuous seconds within that specific location. In other words, if the smartwatch receives Bluetooth low energy signal of a beacon corresponding a location for 40 seconds, the occurrence of that location increases by one unit. Figure 3 (a and b) shows total occurrences of patients in various nursing facility locations (daily) normalized by the number of patients in each category. Darker colors indicate higher frequency of patients visiting a particular location. In short, patients in outcome category “home” traveled within the facility (resident and therapy area) much more frequently than patients eventually admitted to a longer-term care or the “hospital” group. Additionally, no patient in the hospital category used upper body exercise (SciFit), Endorphin, and stair equipment in the therapy area.

## Discussion

### Overview

To the best of our knowledge, this paper and what we described in [13] are first to explore a combination of indoor localization and physical activity tracking to assess older residents. Following baseline investigations [13], in this paper, we highlight significant findings in longitudinal analyses of clinical and sensor-based features.

### Activity With Therapist Versus Resident Time Alone and the Value of Indoor Localization

One of the principal findings of this study is that the energy intensity spent in therapy sessions, unlike in resident room, tend to have similar values in both outcome groups, more significantly toward the end of the rehabilitation period (Figure S1 in Multimedia Appendix 1). Perhaps the therapists in both patient groups are encouraged to complete their therapy activities and are part of an individually designed therapeutic program that aimed to improve functional activity. Moreover, energy intensity spent in the resident room is very similar to overall energy intensity in that patients generally spend most of their time in the resident room. Resident room activity levels are likely to be crucial in determining the outcome of patients, even at early stages of their rehabilitation. Further understanding of the therapeutic skills learned during therapeutic intervention and carryover into the resident room warrants further study.

Based on Table 3, the PT and OT features investigated in this study all improved over time along with the sensor-based feature, energy intensity. However, improvements are more distinguishable between admission day and weeks 1 and 2. On week 3, the mean value for sensor-based features such as overall energy intensity declines. Similarly, OT and PT features show less change compared to week 1 and admission day. One possible reason could be the drop in sample size after week 2 as patients are likely to be discharged earlier. Note that despite the steady PT and OT functional scores in later times, the interquartile range decreases over time, which indicates less variations in functional levels. This could mean that residents achieved their functional goals or plateaued in functional progression. Other aspects that limit a resident’s functional ability need to be examined to determine if nonmotor parameters are limiting a resident’s progress. Cognition, vision, and psychological factors are some of the areas that may limit functional progression.

Table 3 also shows that except the “gait distance in feet,” the improvement of features was not evident after the 2nd and 3rd week. Further exploration of therapy treatment intensity or type of intervention is warranted. Significant improvements in “gait distance in feet” suggest the importance of this feature in clinical assessment. The rest of the gait measures showed they were less likely to change over time. Dynamic gait parameters and their relation to mobility in daily activities need more investigation.

### Sensor-Based Features and Changes in Clinical Assessments

The captured sensor-based longitudinal changes such as lying down, sitting, and overall energy intensity reflect changes in PT and OT features (Table 4). This finding confirms the benefit of remote patient monitoring systems as adjunct tools to further reveal patients’ daily story lines. Such systems can bear valuable information in further understanding the type and intensity of therapy interventions that impact overall functional outcome. Brisk features remained surprisingly unchanged over time when patients were expected to become less sedentary during recovery of functional abilities, at least partially. Average sedentary time among all patients was more than 99.8% and remained unchanged. In other words, the cohort was walking less than 0.2% of the time, measured objectively by the SARP wrist-worn sensor. This finding strongly suggests that focusing on sedentary features among elderly patients is beneficial, confirming the studies in [27-29], contrary to the emphasis many patient monitoring systems place on using activity trackers to count steps [30,31]. This study shows the importance of translating all movements into measurements such as energy, or energy intensity, rather than solely relying on steps. This may shed light on the type of intervention needed for improving the mobility of the elderly resident population.

### Study Limitations

This study had some limitations. Wrist-worn accelerometers used for activity recognition are popular due to their ease of use and ability to capture a comprehensive set of activities. However, interpreting users’ data in sedentary positions such as sitting or standing can be quite challenging. Movements (or lack thereof) in sedentary positions are hard to be distinguished by wrist-worn sensors [32]. Compliance to technology is another obstacle faced in this study. Patients accepting to use the technology is a challenge expected to be generic and present in similar studies.

Battery consumption of smart watches can be problematic when trying to transmit data, hourly or daily. Battery lifetimes are normally insufficient in almost all smartwatch manufacturing brands. Their operating systems are designed to perform sophisticated tasks, many of which are not needed for patient remote monitoring such as receiving messages and calls. Furthermore, consumer-grade wearables have wide variability in their accuracy across a range of functional activities depending on their placement, the individuals’ movement characteristics, speed of walking, using assistive devices, and so on. The best way to tackle this problem is to use wearable sensors specifically designed (hardware and software) for patient monitoring. However, commercially available research-grade sensors are very expensive and not yet clinician and patient friendly [33].

The study cohort had two outcome groups that were not equally presented. The data set predominantly comprised majority class instances and contained only a few instances of patients who were re-admitted to a long-term care. Akin to most imbalanced medical data sets, analyzing such data poses a great challenge [34].

### Conclusions

This study aimed to show that wearable activity trackers, despite raising concerns about their efficacy in quantifying residents’ health, can result in a better understanding of patients’ well-being when tailored for a specific cohort. Such studies can hopefully pave the way in early prediction of hospitalization, developing intervention alerts and improving overall quality of care. As discussed, our remote patient monitoring system, SARP, captures a combination of indoor localization and physical activity features. SARP information on daily and longitudinal activity patterns can be incorporated into mobile health technology platforms to provide a better assessment of underrepresented, particularly frail, populations.

## Appendix

Multimedia Appendix 1 Energy intensity averaged per days in 21 days. Note, the numbers shown on top of the point plots indicate the sample size on the corresponding day specified on the x-axis. Overall energy intensity and energy intensity in resident room and therapy room all improve over time, except at week 3, with a drop in sample size from 83 to 57 participants.

## Abbreviations

ADL: activities of daily living
OT: occupational therapy
PT: physical therapy
SARP: Sensing At-Risk Population
