# Supplementary figures and images for: Physical Activity Behavior of Patients at a Skilled Nursing Facility: Longitudinal Cohort Study
Source: JMIR Mhealth Uhealth. 2022 May 23;10(5):e23887. doi: 10.2196/23887 (PMC9171595; doi:10.2196/23887)

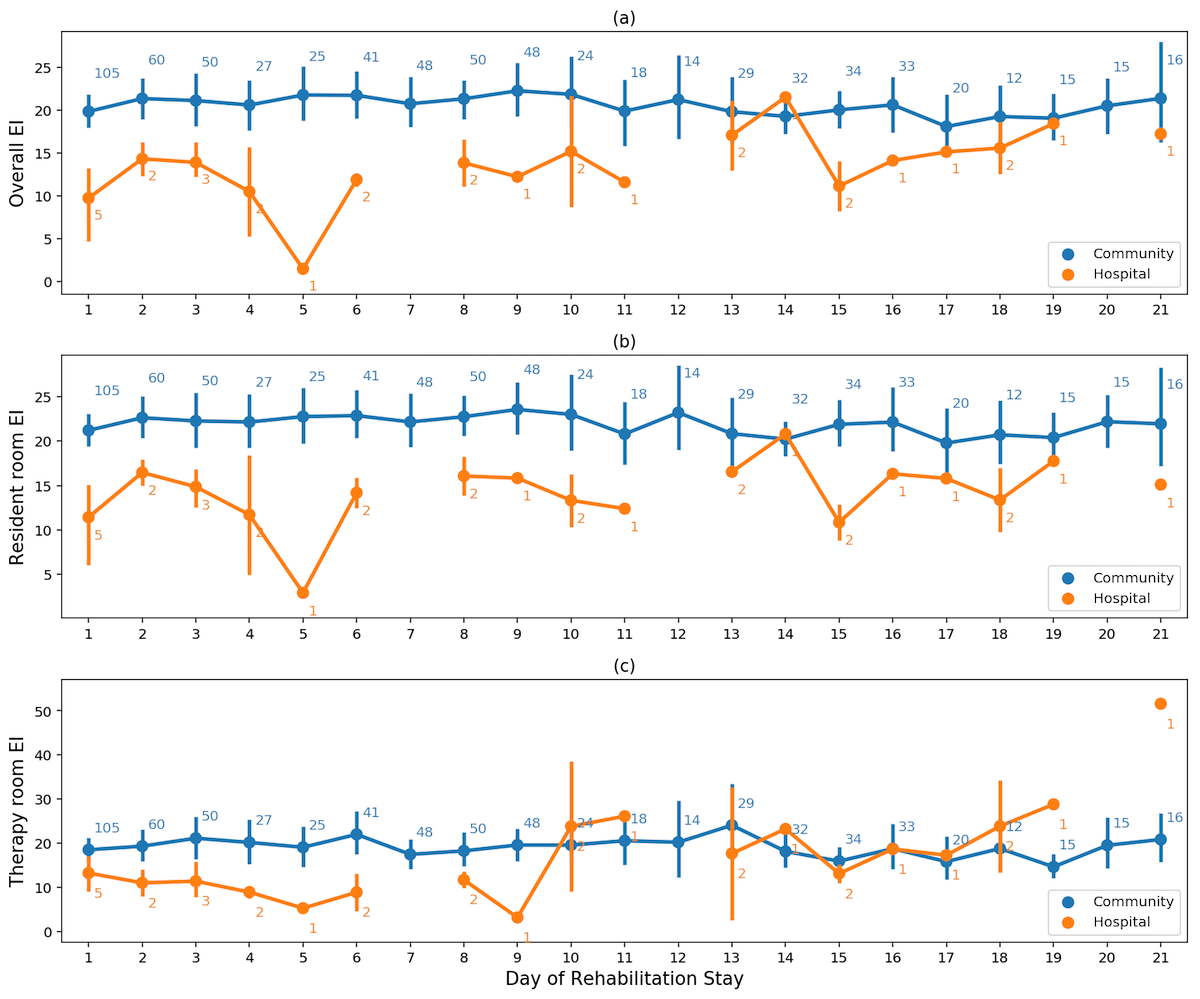

Supplement: Multimedia Appendix 1 [file mhealth_v10i5e23887_app1.png]
